# Differential Sensitivity of Two Endothelial Cell Lines to Hydrogen Peroxide Toxicity: Relevance for In Vitro Studies of the Blood–Brain Barrier

**DOI:** 10.3390/cells9020403

**Published:** 2020-02-10

**Authors:** Olufemi Alamu, Mariam Rado, Okobi Ekpo, David Fisher

**Affiliations:** 1 Department of Medical Bioscience, University of the Western Cape, Bellville, Cape Town 7530, South Africa; olufemialamu@gmail.com (O.A.); maryamadem99@gmail.com (M.R.); oekpo@uwc.ac.za (O.E.); 2 Anatomy Department, Ladoke Akintola University of Technology, Ogbomoso 210241, Nigeria

**Keywords:** oxidative stress, blood–brain barrier, bEnd5, bEnd.3, glutathione, viability

## Abstract

Oxidative stress (OS) has been linked to blood–brain barrier (BBB) dysfunction which in turn has been implicated in the initiation and propagation of some neurological diseases. In this study, we profiled, for the first time, two endothelioma cell lines of mouse brain origin, commonly used as in vitro models of the blood–brain barrier, for their resistance against oxidative stress using viability measures and glutathione contents as markers. OS was induced by exposing cultured cells to varying concentrations of hydrogen peroxide and fluorescence microscopy/spectrometry was used to detect and estimate cellular glutathione contents. A colorimetric viability assay was used to determine changes in the viability of OS-exposed cells. Both the b.End5 and bEnd.3 cell lines investigated showed demonstrable content of glutathione with a statistically insignificant difference in glutathione quantity per unit cell, but with a statistically significant higher capacity for the b.End5 cell line for de novo glutathione synthesis. Furthermore, the b.End5 cells demonstrated greater oxidant buffering capacity to higher concentrations of hydrogen peroxide than the bEnd.3 cells. We concluded that mouse brain endothelial cells, derived from different types of cell lines, differ enormously in their antioxidant characteristics. We hereby recommend caution in making comparisons across BBB models utilizing distinctly different cell lines and require further prerequisites to ensure that in vitro BBB models involving these cell lines are reliable and reproducible.

## 1. Introduction

The blood–brain barrier (BBB) is a functional and morphological interface between the systemic blood circulation and the CNS. The central regulatory component is the brain capillary endothelial cell (BEC), which is assisted by a number of cellular entities, viz. pericytes and astrocytes, together forming an interface, described as the neuro-vasculo-glial unit (NVU). The NVU has the dynamic ability to respond to the homeostatic changes in the brain interstitium ensuring a stable environment for neuronal functionality [1]. From a research point of view, it is therefore of interest to understand the capability of the BEC to withstand oxidative stress (OS), and to scrutinize the BBB in vitro models used to study these physiological processes. However, from a therapeutic point of view the strict regulatory mechanisms for molecules to cross the barrier provide a serious clinical challenge to molecules of desired therapeutic interest to reach their neural target sites [2,3,4,5]. 

Endothelial dysfunction-mediated vascular diseases such as BBB dysfunction have been well linked to excess production of reactive oxygen species (ROS) [6,7]. A major source of ROS in these conditions is the upregulation of nicotinamide adenine dinucleotide phosphate oxidase (Nox, especially Nox2) activity [8,9]. Excess superoxide (O_2_^−^) produced primarily from increased Nox activity undergoes both spontaneous and enzymatic dismutation by superoxide dismutases (SOD), resulting in increase and/or accumulation of its dismutation product, hydrogen peroxide (H_2_O_2_) [10]. Keeping vascular endothelial cells in redox balance involves the activities of several endogenous antioxidants whose activities may be enzymatic or non-enzymatic [10]. Superoxide dismutase (SOD) is an enzymatic, first-line, intracellular antioxidant that catalyzes the conversion of superoxide to hydrogen peroxide [11]. Other important enzymatic antioxidants such as glutathione peroxidase (GPx), and catalase (CAT), help to neutralize H_2_O_2_ to water and oxygen [11]

Endothelial nitric oxide synthase (eNOS) is another enzyme which normally plays a protective role in the vascular endothelial cell through its production of nitric oxide (NO) while oxidizing its substrate L-arginine to L-citrulline using molecular oxygen (O_2_) [12,13]. However, parallel upregulation of eNOS and Nox as occurs in many vascular diseases results in eNOS uncoupling with subsequent conversion of eNOS to an O_2_^−^-producing enzyme that contributes to further vascular oxidative stress [14,15,16]. Furthermore, free radicals such as superoxide can attack polyunsaturated fatty acids (PUFAs) such as arachidonic acid in the cell membranes of vascular endothelial cells to yield advanced lipid peroxidation end products (ALEs) [17]. One of ALEs of relevance is 4-hydroxynonenal (4-HNE), an electrophile that is highly reactive towards nucleophilic thiols and amino groups [17,18]. It readily reacts with proteins (4-HNE-protein adduction), lipids, and nucleic acids of DNA. Its reaction with proteins modifies their activity thus acting as a second messenger in various biologic activities including modification of enzymatic actions or transcription factor modulations that determine cell survival or death [18]. Oxidative stress (OS) occurs in the cells when an unbalanced accumulation of ROS exists within the cell and/or in its immediate environment [19]. BBB dysfunction in relation to OS has been implicated in the initiation and propagation of several neurological conditions such as epilepsy, stroke, and degenerative neuropathies such as Alzheimer’s disease, Parkinson’s disease, and multiple sclerosis [20,21]. Thus, OS has been scientifically documented as a common factor to both the etiologies of these neurological disorders as well as abnormal BBB function. Cell modeling has, to date, provided a robust tool in the in vitro study of the BBB [22,23]. The brain microvascular endothelial cells are the principal cells of the BBB which have had several cell models characterized for use as in vitro models in the study of the BBB [24]. This study focused on two mouse-derived cell lines, b.End5 and bEnd.3, established for use as in vitro models of the BBB [25,26]. It is of interest to understand how the endothelial cells of the BBB respond to oxidative stress as well as the endothelial cell-specific events that underlie the abnormalities of BBB permeability. It is, however, disconcerting that most of the in vitro cell models in use for BBB studies have not been characterized for their oxidative/antioxidant features, which are necessary for the definition of OS, as well as for features that characterize changes in oxidative stress responsible for the observation of abnormal permeability under OS conditions. In this study, we profiled for the first time, b.End5 and bEnd.3 cells, both mouse-derived cell lines obtained after immortalizing primary mouse brain endothelial cells by infection with middle T antigen-expressing polyoma virus, for their resistance against a suitably-selected ROS, hydrogen peroxide (H_2_O_2_), which can permeate all intracellular membranes and thus exert its effects on organelles [27]. Cellular glutathione content (reduced/oxidized form [GSH/GSSG]), a well reported marker of cellular oxidative status, is a tripeptide, L-γ-glutamine-L-cysteinyl-glycine, that acts as an endogenous cellular antioxidant either by direct neutralization of ROS or as cofactor for the antioxidant enzyme, glutathione peroxidase (GPx) [28]. [Hereafter, we refer to the full component of reduced and oxidized glutathione as ‘total glutathione while GSH and GSSG refer to the ‘reduced glutathione’ and ‘glutathione disulfide/oxidized glutathione’ fractions respectively]. This functional capability of GSH is conferred by its active thiol group residing in its cysteine residue [29]. Glutathione exists within cells in either the reduced (GSH) form or in oxidized form as glutathione disulfide (GSSG). It is usually synthesized as GSH but oxidized to GSSG upon participating in a redox reaction. Changes in cellular reduced glutathione content as well as changes in cell viabilities were used to assess cellular capacities to respond to varying levels of ROS. This protocol is robustly useful whenever it is desired to use these cells to study oxidant effects on the BBB. 

## 2. Materials and Methods

### 2.1. Bio-Reagents

Analytical grade reagents were used for all experiments. These included monochlorobimane (Molecular Probe M1381MP, Eugene, OR 97402, USA), trypan blue, (Gibco 1520-061, Gaithersburg MD 20877, USA), tris (2-carboxyethyl) phosphine hydrochloride, TCEP (Sigma C4706, Laramie, WY 82070, USA), GSH-Glo^TM^ Glutathione Assay Kit (Promega V6911/2, Madison, WI 53711, USA), Trolox ((±)-6-Hydroxy-2,5,7,8-tetramethylchromane-2-carboxylic acid, Sigma 238813, Laramie, WY 82070, USA), Cell Proliferation Kit II (XTT) (Roche, Sigma 11465015001, Laramie, WY 82070, USA), and hydrogen peroxide (30%, Merck Millipore 107209, Feldbergstraße 80, 64293 Darmstadt, Germany).

### 2.2. Cell Cultures

Two mouse brain endothelioma cell lines (b.End5 and bEnd.3) were used in this study. The b.End5 (ECACC 96091930, Salisbury, Wiltshire SP4 0JG, UK) cells were cultured in Dulbecco’s modified Eagle’s medium (DMEM Lonza BE 12-719F, Salisbury, MD 21801, USA.) supplemented with 10% fetal bovine serum (FBS Biowest12010S181G, Rue du Vieux Bourg, 49340 Nuaillé, France), 100 U/mL penicillin/streptomycin (Lonza DE17-602E), 1 mM sodium pyruvate solution (Lonza BE13-115E), and 1% non-essential amino acids (NEAA Lonza BE13-114E) at 37 °C and 5% CO_2_ in a humidified incubator. For all experiments, b.End5 cells at passages 5–20 were used and culture medium was changed every 2–3 days. Prior to experimentation, cells were rinsed in 1× phosphate buffer solution (PBS). The adherent cells were detached by the addition of 0.25% trypsin-EDTA after which equal volume of fresh media was added and the cell suspension aspirated into 15 mL conical tubes. The cell suspensions were then centrifuged for 5 min at rpm of 2500 to obtain a cell pellet. The supernatant was then removed by gentle aspiration and thereafter, 5 mL of fresh media added to bring the cell pellets back in suspension. The bEnd.3 (ATCC^®^ CRL-2299, Gaithersburg, MD 20877, United States ) cells were cultured in Dubelcco’s Eagle’s medium (Gibco 11320074) supplemented with 10% fetal bovine serum (FBS Biowest S12010S181G), 100 U/mL penicillin/streptomycin (Lonza DE17-602E), and L-glutamine to a final concentration of 4.1 mM (Invitrogen 25030081, Camarillo, CA 93012, United States ) and at 37 °C and 5% CO_2_ in a humidified incubator. The bEnd.3 cells used for all experiments were at passages 5–20 and the medium was changed every 2–3 days. Prior to cell seeding for experiments, adherent bEnd.3 cells were similarly brought to suspension as described for the b.End5 cells.

### 2.3. Hydrogen Peroxide Treatments

A stock solution of H_2_O_2_ (9.8 M, Merck Millipore, Feldbergstraße 80, 64293 Darmstadt, Germany) was diluted to the required concentrations in complete media. Cultured b.End5 and bEnd.3 cells were divided into labelled treatment wells exposed to H_2_O_2_ concentrations ranging between 10 µM and 2 mM and cells that were unexposed to H_2_O_2_ served as control. To determine the antioxidant capacity of cultured cells both b.End5 and bEnd.3 cells were seeded in separate transparent 96-well plates at 1 × 10^4^ cells per well in 200 µL of normal media and allowed to attach overnight. Media were then aspirated and replaced with either 100 µL of fresh media to serve as control or 100 µL of media dosed with the appropriate concentrations of hydrogen peroxide for another 24 h. Experiments were repeated thrice and average values of parameters were recorded. Viability of the cells exposed to different concentrations of hydrogen peroxide were then analysed and compared against viability measures for the control cells under same duration in culture. 

### 2.4. Viability Assay

Cells were cultured in a transparent 96-well plate as described for hydrogen peroxide treatments. Equal numbers of b.End5 cells were seeded into eighteen treatment (H_2_O_2_) wells (*n* = 3) starting from control (unexposed) and treatment with [H_2_O_2_] in multiples of 50 µM up to a maximum of 850 µM. For cultured bEnd.3 cells, equal numbers of cells were seeded into sixteen sets of 3 wells (*n* = 3) and treated as control (unexposed), then [H_2_O_2_] in multiples of 10 µM up to 100 µM and then in multiples of 100 µM up to a maximum of 500 µM. A blank column of three wells was also included in both treatment plates to facilitate the determination of relative absorbance units. The XTT [30] viability assay kit (Roche) was used to quantify cell viability after treatment for 24 h. The XTT reagent was reconstituted by mixing 100 µL of electron-coupling reagent (0.383 mg/mL) with 5 mL of XTT labelling reagent (1 mg/mL) to activate it as per manufacturer′s recommendation. Reconstituted XTT, 50 µL, was then added to each well containing 100 µL of cell culture and incubated for 4 h at 37 °C in a CO_2_ incubator. Absorbance was then read for each well at 450 nm and blank-corrected values obtained using a GloMax–Multi Detection System (Promega, Madison, WI 53711, USA). The absorbance measures directly correlated with the viability of the cells in each well.

### 2.5. Fluorescent Detection of Glutathione in Cultured Cells

Equal numbers of b.End5 and bEnd.3 cells were cultured under standard conditions on microscopic glass slides in separate Petri dishes. The cells were then allowed to attach overnight in all Petri dishes and cells on each slide were used to demonstrate glutathione. Briefly, the medium was removed from the attached cells and were rinsed twice with PBS solution, pH, 7.4, and then incubated with monochlorobimane solution (mBCl, Molecular Probe^TM^ M1381MP) 60 µM in complete DMEM for 30 min [31]. Following mBCl loading, slides were fixed using a mixture of 4% paraformaldehyde (PFA) and 0.2% glutaraldehyde (GA) in PBS solution at pH 7.4 for 10 min and following fixation, cells were nuclear-counterstained by incubating slides with 20 µg/mL propidium iodide (PI) solution for 15 min. DABCO (1,4-diazobicyclo-[2,2,2]-octane) mountant, 20 µL, was added to each slide mounted with cover slips. Cells on each slide were then viewed and imaged under a Nikon Eclipse 50i fluorescent microscope at λ_ex_/λ_em_ of 365/490 nm and 439/636 nm for mBCl and PI, respectively.

### 2.6. Quantification of Total Cellular Glutathione in bEnd5 Cells

To accurately quantify the total amount of glutathione in a single b.End5/bEnd.3 cell, we used a GSH-Glo™ Glutathione Assay Kit which works by a luminescence assay to detect and quantify glutathione [32]. The assay is based on the conversion of a luciferin derivative into luciferin in the presence of glutathione, catalyzed by glutathione-S-transferase (GST). The reaction is further coupled with a firefly luciferase which leads to the generation of luminescence signal proportional to the amount of glutathione in the sample. To estimate glutathione fairly accurately in 1 × 10^4^ cells, according to manufacturer’s recommendation and to control for cell proliferation occurring alongside cell attachment, cells were plated in white 96-well plates and incubated at 37 °C and 5% CO_2_ at a density of 4 × 10^3^ cells per well for the b.End5 cells and 4.5 × 10^3^ cells per well for the bEnd.3 cells, based on an optimized number of the respective cells that gave the target density at 24 h in culture (based on our data from proliferation study, not shown). Cells were plated in columns of four wells (*n* = 4) in a 96-well white bottom plate and 100 µL of prepared 1X GSH-Glo reagent was transferred to each well. In order to measure the total glutathione (GSH + GSSG), 100 µL of 1 mM tris (2-carboxyethyl) phosphine (TCEP) was added to a group of four wells in addition to the GSH-Glo reagent according to the GSSG recycling method [33]. The contents of the wells were agitated briefly on an orbital shaker before incubation at room temperature for 30 min. Then, 100 µL of reconstituted luciferin detection reagent was transferred to each well, and the plate was mixed briefly on an orbital shaker before incubation at room temperature for 15 min. Luminescence values were then read using a GloMax–Multi Detection System (Promega, Madison, WI 53711, USA). Luminescence readings were converted to GSH concentration using a standard curve generated from a 5 mM GSH standard supplied by the manufacturer.

### 2.7. Statistical Analysis

Statistical analysis was done using Graph Pad Prism.5 statistical analysis software. Data were expressed as mean ± SEM and significant differences in data were accepted at *p* < 0.05

## 3. Results

In this study differences in antioxidant capacities of two brain endothelial cell lines from the same animal species were observed. We firstly established baseline data for both cell lines in terms of their endogenous glutathione concentrations, and secondly, we profiled the response of these cell lines to an exogenous ROS stress, H_2_O_2_, with respect to the content of the endogenous antioxidant, glutathione, using fluorescent imaging, and fluoro-spectrometric quantification.

### 3.1. Both b.End5 and bEnd.3 Cells Demonstrated Glutathione Presence on Fluorescent Microscopy

**Figure 1:**
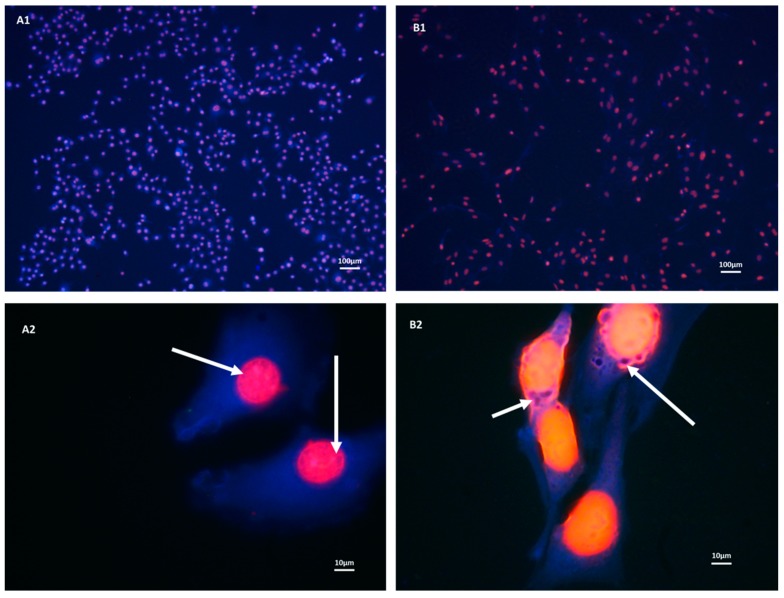
Micrographs show fluorescent images of b.End5 (**A1**) and (**A2**) and bEnd.3 cells (**B1**) and (**B2**) in normal culture. Both cells showed blue monochlorobimane solution (mBCl) fluorescence (due to binding with reduced glutathione (GSH)) in their cytoplasm, though at the higher magnification b.End5 appeared more deeply stained. Furthermore, plate A2 revealed a lower nucleo-cytoplasmic ratio in b.End5 cells suggesting more cytoplasmic GSH content than in bEnd.3 cells. Furthermore, multiple segments and rings of blue fluorescence (white arrows) were indicative of glutathione observed within the nuclei and nucleo-cytoplasmic interface in the cells of both cell types (Plates A2 and B2).

We first investigated presence and distribution of glutathione in each of the selected cell lines. Monochlorobimane with propidium iodide nuclear counterstaining revealed blue fluorescence due to the presence of glutathione while the nuclei fluoresced red (Figure 1). Both cells appeared intensely fluorescent for glutathione, however, bEnd.3 showed less cytoplasmic glutathione possibly due to a higher nucleo-cytoplasmic ratio. Variable segments and rings of blue fluorescence were observed around the periphery of the nuclei in both cells (Figure 1, Plates A2 and B2) which are evidence of glutathione presence within the nuclear structure. This was more prominently observed at the nucleo-cytoplasmic interface in both cell types and especially in the bEnd.3 cell (Figure 1, Plate B2). 

### 3.2. Glutathione Contents of Both b.End5 and bEnd.3 Cells Are Comparable in Normal Culture

**Figure 2:**
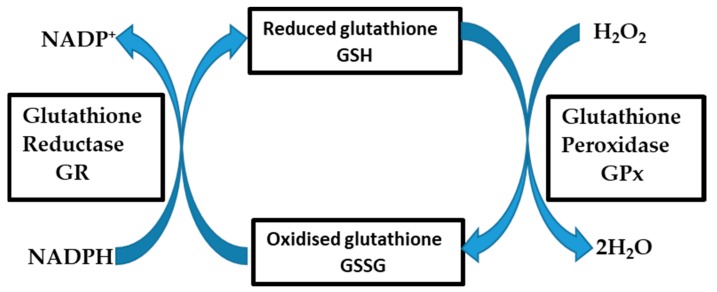
The above diagram illustrates the redox buffering reaction of the glutathione system. Glutathione peroxidase (GPx) enzymatically converts H_2_O_2_ to 2H_2_O using reduced glutathione (GSH) as substrate which is then converted to its oxidized form, glutathione disulfide (GSSG) in the process. The GSSG in a second reaction involving glutathione reductase enzyme is converted back to GSH and thus GSH is recycled. The glutathione reductase reaction contributes significantly to the cellular maintenance of pooled reduced glutathione for redox defense.

The difference in blue fluorescence between the two cell types provided micrographical evidence for the presence and distribution of glutathione. The objective quantification of the levels of glutathione in each cell type was required to compare the cells’ ability to respond to OS (Figure 2). 

**Figure 3:**
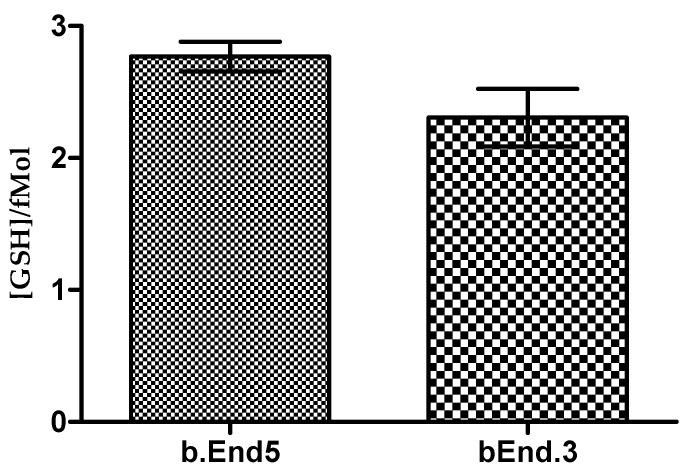
A comparison of GSH concentration in the two types of cells revealed no statistical difference between the two means (Student′s *t* test: *p* = 0.1325).

Total and reduced fraction of the glutathione content in both cell types were experimentally determined while the content of oxidized glutathione was derived by deduction of the reduced glutathione values from the respective total glutathione values. The value of the total glutathione was obtained by reducing the GSSG fraction in each sample using TCEP which also has the ability to recover protein-bound GSH. However, because GSH exists within cells either freely or bound to proteins the recovered GSH still constituted a fraction of the total glutathione pool. In b.End5 cells the amount of glutathione per unit cell was higher than that of bEnd.3, however, the difference was not statistically significant (*p* = 0.1325) (Figure 3). Estimated values were 2.769 ± 0.113 fM/cell for b.End5 and 2.305 ± 0.219 fMol/cell for bEnd.3. Corresponding GSSG values for both b.End5 and bEnd.3 cells were respectively 0.139 ± 0.006 fM/cell and 0.115 ± 0.011 fM/cell. GSH/GSSG ratio in both cells approximated to 95%/5%.

### 3.3. Antioxidant Capacity Is Higher in b.End5 Cells

**Figure 4:**
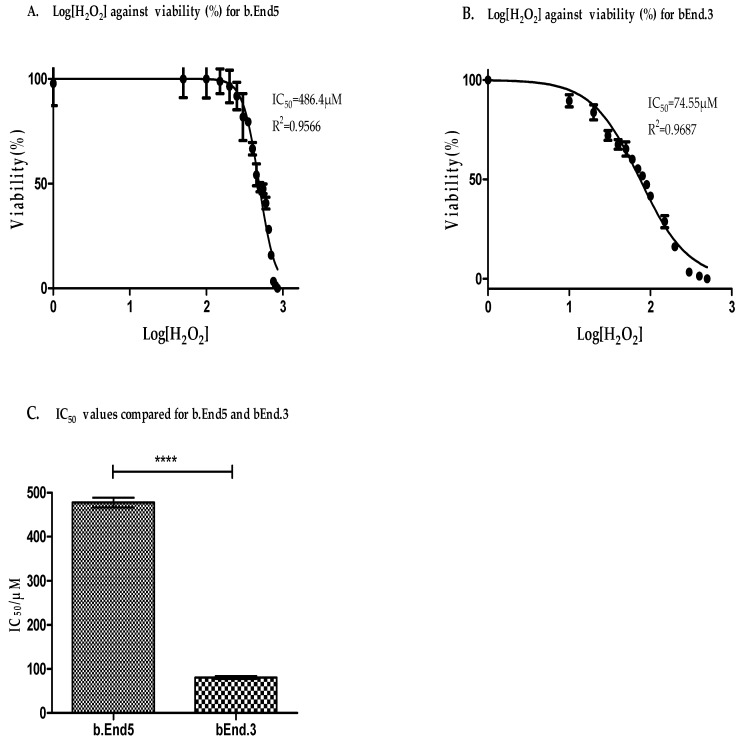
A non-linear regression analysis of logarithmic values of [H_2_O_2_] against normalized viability was used to determine the hydrogen peroxide concentration that caused 50% inhibition of cell viability in b.End5 cells. Cells were exposed to [H_2_O_2_] that ranged between 0 (control) and 850 µM for 24 h in flat-bottom transparent 96-well plates. Viability changes correlated to the absorbance measured at 450 nm from each well following incubation with XTT reagent for 4 h. (**A**) Results showed IC_50_ for b.End5 cell as equivalent of 486.4 µM at r^2^ = 0.9566. (**B**) Data for bEnd.3 cells was obtained by exposing cultured bEnd.3 cells to [H_2_O_2_] ranged from 0 (control) to 500 µM and non-linear regression analysis done as described above. Results showed IC_50_ for bEnd.3 cell to be 74.55 µM at r^2^ = 0.9687 µM. (**C**) Graph of the IC_50_ values for the b.End5 compared with the same values for the bEnd.3 cells (annotation * denotes statistically significant difference between the values shown). IC_50_ values for b.End5 and bEnd.3 cells were statistically compared using the Student′s *t* test. The analyzed data showed that the IC_50_ value was significantly higher for b.End5 cell than for the bEnd.3 cell (*p* < 0.0001).

Although both cell lines tested showed that glutathione was abundantly present in both the cytoplasm and nucleoplasm, the response of these cell lines to ROS stress remains to be investigated. Both bEnd.3 and b.End5 cells treated with increasing concentrations of hydrogen peroxide showed changes in viability which correlated with the relative absorbance units obtained from XTT proliferation assay. The assay is based on the cleavage of the yellow tetrazolium salt, XTT, to form an orange formazan dye by mitochondrial dehydrogenases in metabolically active cells. Because an increase in the number of cells results in an increase in the overall activity of mitochondrial dehydrogenases in the samples, these changes correlated to the amount of orange formazan formed, a parameter that was monitored by the relative changes in the absorbance. The viability changes in both cells were normalized as percentages of the control, unexposed, cells and plotted against the logarithmic values of the various concentrations and the half-maximal inhibitory concentrations (IC_50_) were determined and compared for the two cells (Figure 4A,B). Experiments were repeated three times and average values were analyzed. Results showed that the IC_50_ for the b.End5 cell was significantly higher than for the bEnd.3 cell (Figure 4C). 

### 3.4. Glutathione Was More Resistant to Oxidant Depletion in b.End5

**Figure 5:**
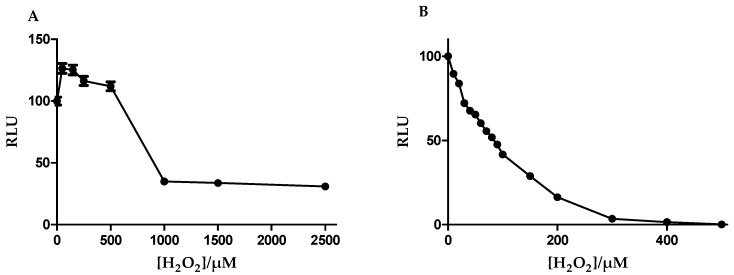
(**A**) and (**B**) show trends in the glutathione contents of b.End5 and bEnd3 cells with exposure to increasing [H_2_O_2_]. Cells were exposed to increasing concentrations of [H_2_O_2_] for 24 h and average cellular glutathione contents estimated using the GSH-Glo kit. Glutathione contents of the cells correlated directly to the relative luminescent values (RLU) obtained following incubation with the optimized reagents of the GSH-Glo assay kit described. Data in Figure 4A represents b.End5 cells and shows an upward trend in the glutathione content of the b.End5 cells upon exposure to [H_2_O_2_] of 0–250 µM. Above this concentration was observed a downward trend though values remained higher or at par with the starting point until [H_2_O_2_] higher than 500 µM. From this point a steady decline occurred until about 1 mM [H_2_O_2_] followed by a plateau but not a complete depletion. Data in Figure 4B represent the trend in bEnd.3 glutathione changes with increasing [H_2_O_2_]. A steady decline was observed until complete depletion at about 400 µM [H_2_O_2_].

Changes in the glutathione content of equal number of cells were plotted against the hydrogen peroxide concentrations. This allowed for the analysis of the physiological response (endogenous antioxidant response) of the two cell lines to increased H_2_O_2_ concentrations. The profile in glutathione depletion against increasing concentrations of hydrogen peroxide for the two cell lines was examined (Figure 5A,B). The data showed a steady decline in the glutathione content of the bEnd.3 cells (Figure 5B) while the b.End5 cells, in contrast, showed an initial significant increase in glutathione content and then a decline (Figure 5A). In b.End5 cells, glutathione increase was sustained either higher or at par with the control cells until about a concentration of 500 µM which was close to its IC_50_ value (Figure 5A). Also, the glutathione content of b.End5 cells were observed to decline to a steady lowest value at about 1 mM hydrogen peroxide concentration and thereafter remained constant. 

## 4. Discussion

Although several previous reports have reported the existence of strain-specific genes in contributing to some differences in the physiological characteristics of different organs and/or systems in animals of the same species [34,35,36], researchers have largely ignored this evidence in choosing cell lines to model a physiological system. This is particularly evident in the use of cell lines modelling the BBB, where a variety of primary and immortalized cell lines have been used to study its physiological functions. This phenomenon creates un-necessary murkiness in comparing data between different studies. In this study, we were able to illustrate convincingly that differences in the antioxidant characteristics of the two cell lines (b.End5 and bEnd.3) (Figure 4A–C) of the same species, have distinctly different response profiles to an escalating ROS stressor. These two cells were derived from primary brain endothelial cells of two different mouse strains, BALB/c and SV129 respectively, by infection with a retrovirus coding for the Polyoma middle T-antigen [37,38]. Both have been used extensively to study BBB function [39,40,41], all with the underlying assumption, that there is physiological parity between these cell lines. 

We investigated the cellular content of the glutathione antioxidant in the two cell types under normal culture conditions (as per the instructions provided by the suppliers), first by fluorescent microscopy, and although we easily established the cellular presence for glutathione, we were unable to distinguish clear quantitative differences between the two cell lines. Close examination of the fluorescence micrographs clearly illustrated the even distribution of glutathione throughout the cytoplasm of both cell types, however, the higher ring of fluorescence just inside the nuclear membrane seem to suggest that glutathione plays an important role in neutralizing ROS entering into the nucleus. Given that DNA fragmentation is prone under conditions of OS [42,43], this provides solid circumstantial evidence as to the role of glutathione in protecting the nuclear material from ROS compromise. Although fluorescence demonstrated the presence of glutathione in both types of cells, the presence of blue fluorescence provided subjective evidence of increased cytoplasmic glutathione within b.End5 cells as evidenced by a lower nucleo-cytoplasmic ratio in the b.End5 cells with consequently more cytoplasmic content of glutathione. 

Our quantitative data of the glutathione content of the b.End5 and bEnd.3 cell types substantiated our visual observations and, although b.End5 cells had a slightly higher mean glutathione content, statistically no significant difference in glutathione quantity was found between the respective cell lines (Figure 3). Calculations from data indicated that the GSH concentration per unit cell for the b.End5 and bEnd.3 cell types were within the range of 2.769 ± 0.113 fMol and 2.305 ± 0.219 fMol respectively. Both cell types also have GSH/GSSG ratio of approximately 95%/5% which indicated that both cells were redox-stable in normal culture. Comparisons of the average cellular glutathione per single cell for both the b.End5 and bEnd.3 cell lines predict that these cells are well suited for OS resistance in that their GSH contents are in the range of cells specialized for detoxification, such as the liver cancer cell line, HepG2 with GSH content of 2.9 fMol/cell [44]. These data, taken independently endorses the use of these cell lines for BBB modeling. It is presumptuous to assume that simply on the basis that the cell lines have similar basal levels of endogenous antioxidants that these cells may indeed respond to incremental ROS stress in a similar manner. This is all the more complicated given the arbitrary concentrations of ROS stressors used in experiments to demonstrate OS on the BBB [45,46]. 

To evaluate the response of the endogenous antioxidant glutathione to incremental concentrations of H_2_O_2_ in each cell-line, we then determined the relative levels of OS that causes decompensation of the GSH/GSSG antioxidant system in the cell after 24 h exposure (Figure 4A,B). In this study we profiled the cell lines using the glutathione system which has been reported as a reliable and sensitive marker of oxidative cellular status [47,48]. Furthermore, because the physiological variable of cell viability was used as a general indicator of the response of the cells to ROS (H_2_O_2_), it is scientifically plausible that viability changes will be a reliable indicator of the total cellular antioxidant capacity, and therefore, a singular marker (glutathione) would provide enough insight on the differences in the total antioxidant capacities of the two cell lines. Using the IC_50_ [49]values for comparison, we documented for the first time evidence of a significantly greater capability of the b.End5 cells to neutralize higher ROS concentrations than the bEnd.3 cells ([*p* < 0.0001] Figure 4C). When we studied the profile in the GSH depletion within the cells exposed to increasing ROS load the data showed that the bEnd3 cell type has a very limited ability for de novo upregulation of glutathione synthesis under condition of increased ROS accumulation (Figure 5B), whereas the b.End5 cell type showed ability to sustain adequate levels of glutathione, to elevate levels of GSH in response to initial low concentration of ROS, and to maintain this sustained endogenous GSH levels despite several subsequent increases in the concentration of ROS (Figure 5A). The mechanism for this divergent reaction of the glutathione system in the two cell types against increasing concentration of ROS is not clear, and requires further study. However, this observed difference is suggestive of inter-strain differences in the system that regulates ROS accumulation within these cells, as has been reported for several diverse physiological characteristics in different strains of cells from the same species of animal [35,49,50]. Differentially expressed genes in specific domains of the system that regulate ROS in intracellular milieu or perhaps strain-specific genetic alteration following viral oncogenic transformation of the primary cells could be responsible [51]. Mouse strain-specific differences in neuro-behavior, neuronal excitability, susceptibility to fibrosis, anti-inflammatory response, and bone density have been previously reported [50]. These previous reports have strengthened our position that strain-specific genes are very important in shaping the phenotypic differences in the two cell types investigated with respect to their glutathione system plasticity to OS. This finding has an important bearing on experimental designs aimed at studying effects of OS on the BBB. However, whether these two mouse cells respond differently to OS in the in vivo situation is not known. We propose here that a genome-wide genetic screen of the cells in use for evaluating the physiology of the BBB will identify important differences in the genes that control several key functions of the BBB [52]. Also, further research is required to determine these properties in primary cells of the mouse brain endothelial cells with the potential for the unraveling of the true behavior of the mouse brain endothelial cells during OS conditions with respect to capacity for ROS neutralization and the glutathione system response.

Given that under control conditions we are confident that the BECs would be in redox balance, it is, therefore, unimportant to localize the source of endogenous ROS production in response to exogenous ROS exposure, neither does it make scientific sense to measure ROS production from the cell organelles when the source of ROS was clearly defined as the experimental treatment (exogenous H_2_O_2_). Exogenous ROS treatment would gauge the physiological capacity of the cells to respond to ROS exposure from an exogenous source. Exogenous exposure would be additional to the normal ROS load generated by normal physiological cellular processes. Thus, the measuring of ROS levels while exposing cells to exogenous H_2_O_2_ in this study would be superfluous. Nevertheless, it might be assumed that the study largely ignored the spatiotemporal localization and quantification of endogenous ROS production, but we are aware of the lack of the use of these techniques, which is intended to be the focus of our future study, in which we propose to measure ROS produced endogenously by cellular organelles using a combination of immuno-electron microscopy as qualitative and ELISA as semi-quantitative methods [53,54,55].

## 5. Conclusions

The glutathione system responses and ROS buffering capacities are clearly linked and they determine the magnitude of ROS that induces OS in the b.End5 and bEnd.3 mouse brain endothelial cell line models of the BBB. Strain-specific differences in the different cells will result in different definitions of OS in different models of different animal strains within the same species. Thus it is important to establish experimental parameters that best define OS for each endothelial cell model of the BBB for reproducibility. Such information will avail researchers of opportunity to verify and select appropriate models of BBB endothelial cells for specific redox investigations and enable them to draw comparable and reproducible conclusions.
